# Postoperative renal failure following inferior vena caval tumor resection with right nephrectomy: A case report and review of literature

**DOI:** 10.4103/0970-1591.38612

**Published:** 2008

**Authors:** B. Satheesan, S. R. Subramaniam, N. Kathiresan, B. Jayanand Sunil

**Affiliations:** 1 Division of Genitourinary Oncology, Cancer Institute (W.I.A), Adyar, Chennai, India; 2 Department of Surgical Oncology, Cancer Institute (W.I.A), Adyar, Chennai, India; 3 Vijay Heart Foundation, Chennai, India

**Keywords:** Inferior vena cava tumors, renal vein occlusion, vascular leiomyosarcoma

## Abstract

Tumors arising from the inferior vena cava are rare. The commonest histological type among these rare tumors is leiomyosarcoma. Complete resection of the tumor is the mainstay of management. Concomitant right nephrectomy during resection may be required. As the collateral circulation of the left renal veins is extensive and well-developed, reconstruction of left renal vein is not required generally. This case report provides a situation, which may warrant the reconstruction of the left renal vein as the patient developed postoperative renal failure. Authors recommend the reconstruction of the left renal vein in a similar situation disregarding the collateral circulation.

## INTRODUCTION

Primary smooth muscle tumor of vascular origin is rare, the commonest being leiomyosarcoma. Though these tumors arise in large arteries also, the incidence in veins is five times more.[1] The commonest site of vascular leiomyosarcoma is the inferior vena cava (IVC). Though the infiltration of contiguous organs by the tumor is rare, the magnitude of the tumor may warrant the resection of the right renal vein and right kidney to obtain tumor-free margins. In general the immediate reconstruction of the left renal vein may not be required as the collateral circulation is adequate for the left kidney.[2] Here we present a case of inferior vena cava leiomyosarcoma (IVCLMS) resected en bloc with right kidney who presented with postoperative acute renal failure leading to chronic renal failure. The left renal vein collaterals appeared adequate to avoid immediate reconstruction of the left renal vein. But the patient developed anuria and she required prolonged dialysis after reconstruction of the left renal vein.

## CASE REPORT

**Figure 1:**
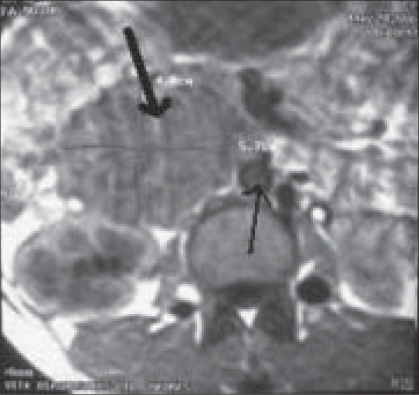
Magnetic resonance imaging of the tumor. Thick arrow – The tumor and the thin arrow – Aorta

**Figure 2:**
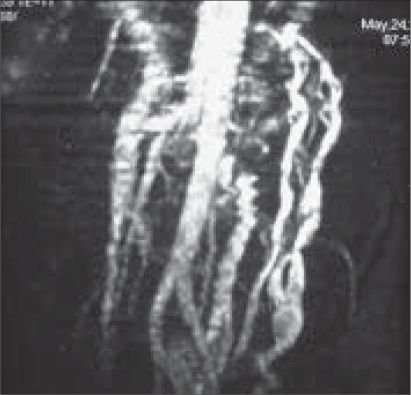
Magnetic resonance angiogram: extensive collaterals formed

**Figure 3:**
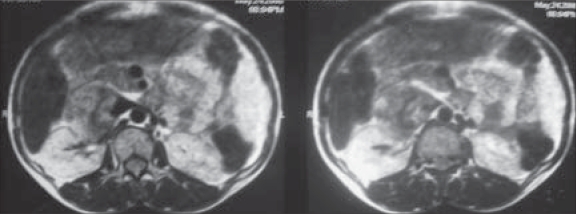
The left renal vein opens into partly patent IVC. The tumor extends below the entry point

**Figure 4:**
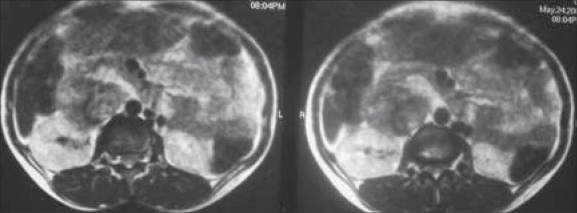
Infra-renal IVC is blocked completely by the tumor

A 25-year-old lady without any co-morbid illness presented in May 2006, with history of nonspecific abdominal pain and backache of one year duration. General condition was unremarkable. There was no pedal edema. Examination of abdomen revealed a 6 × 5 cm nodular retroperitoneal swelling in the right hypochondrium. Radiological imaging with ultrasonography, computed tomography and magnetic resonance imaging and magnetic resonance angiography revealed a retroperitoneal mass arising probably from the IVC, encasing the right renal vein and infiltrating the right kidney with ?tumor in the IVC [Figure 1]. Multiple large collateral vessels were also demonstrated [Figure 2]. The infra-renal IVC was completely blocked by the tumor [Figures 3 and 4].

**Figure 5:**
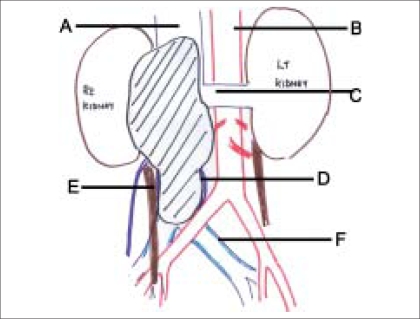
A-IVC, B-Aorta, C-Left renal vein, D-Intraluminal tumor distending IVC, E-Right ureter, F-Thickened common iliac vein. Shaded area is the IVC tumor

She underwent exploratory laparotomy under general anesthesia on 5 July 2006. The tumor was found to be arising from the suprarenal IVC (around 2 cm below the hepatic veins) with dense adherence to right kidney and extension of tumor down to right renal vein and both common iliac veins (whitish discoloration and thickening) [Figure 5]. Left renal vein was free and IVC was patent at its entry. The collaterals were large and well-developed. But the IVC below the renal veins was blocked completely by the tumor which became circumferential. The tumor which extended downwards was intra-luminal in the lowermost part of the IVC. She underwent resection of the infra hepatic IVC distally up to just beyond the bifurcation of both common iliac veins with right nephrectomy and legation of left renal vein. The IVC was resected beyond the bifurcation of common iliac veins because of the suspicious thickening and whitish color of the IVC. In the immediate postoperative period, she developed anuria and azotemia (5 mg/dl of creatinine and 80 mg/dl of urea.) The USG and Doppler study done then showed congested left kidney with sluggish flow in the left renal vein. She was re-explored within 18 h. Intraoperatively the left kidney appeared congested and there was a small clot at the opening of the left gonadal vein. An inter-position PTFE graft was placed between the left renal vein and splenic vein. Her renal output improved gradually and she required multiple sittings of hemodialysis. After six weeks of intermittent hemodialysis, the renal biochemical parameters became steady in the supernormal level indicating the onset of chronic renal failure. (Blood urea between 50-70 mg/dl and serum creatinine between 2.5-4 mg/dl.) She improved symptomatically. She did not develop lower limb edema. The follow-up chest X-ray revealed a solitary pulmonary metastasis in the right lung upper zone after three months. She was advised best supportive care.

Histopathological examination revealed a 9 × 6 × 6 cm gray white tumor arising from the IVC with features suggestive of leiomyosarcoma Grade 2-3. Resection margins were free. The tumor was involving the right renal vein with adherence to the right kidney, but was not infiltrating it. There was no lymph nodal metastasis (lymph node status 0/17).

## DISCUSSION

Primary IVC tumor was first described and published in 1871. The most common smooth muscle tumor of the inferior vena cava is leiomyosarcoma. International IVC leiomyosarcoma registry was established in 1992. Two hundred and eighteen cases have been registered until 1996.[3] The median age group is between 51-59 years with female preponderance.[3,4] The most common location in the IVC is the area between the hepatic veins and renal veins. These tumors are slow-growing tumors providing adequate time for the collaterals to develop. The symptoms are generally nonspecific.[5]

Complete surgical resection forms the mainstay of management. The resection may require reconstruction of IVC or renal veins depending on the extent of collateral development and completeness of IVC obstruction. Perioperative mortality described in the literature ranges from 2.5-20%. One of the major causes of perioperative mortality is acute renal failure.[4] The postoperative renal failure related to venous congestion is usually reversible.

Extended resections of IVCLMS with concomitant right nephrectomy, bilateral nephrectomy and auto transplantation and liver resections have been reported. Reconstruction of IVC is becoming less controversial nowadays with advancements in technology and growing surgical expertise. Reconstruction of IVC may not be needed if the suprarenal and perirenal IVC are completely occluded by tumor and adequate extensive collaterals have developed.[4] But some authors believe that wide resection of the tumor often disrupts the collaterals rendering them insufficient to prevent lower body and limb edema.[4] Concomitant resection of the right kidney and ligation of left renal vein has also been described in a few series without any postoperative renal failure.[4] The left renal vein with major collaterals in the gonad vein, middle capsular vein, adrenal vein and lumbar-azygos veins usually survive the threat of thrombosis and subsequent venous occlusion. In our patient, there was only partial occlusion of IVC at the level of the left renal vein with significant drainage. In similar situations, if the renal vein stump pressure is more than 30-40 mm of water, reconstruction is advised. This measurement is cumbersome. Hence we recommend reconstructing the left renal vein with or without reconstruction of IVC in similar situations.

In a situation of single kidney with inferior vena cava resection, it is advisable to perform the reconstructive surgery. It is especially true if the renal vein and associated segment of IVC are patent.
